# PEDF increases the tumoricidal activity of macrophages towards prostate cancer cells *in vitro*

**DOI:** 10.1371/journal.pone.0174968

**Published:** 2017-04-12

**Authors:** Dalia Martinez-Marin, Courtney Jarvis, Thomas Nelius, Werner de Riese, Olga V. Volpert, Stéphanie Filleur

**Affiliations:** 1 Department of Urology, Texas Tech University-Health Sciences Center, Lubbock, Texas, United States of America; 2 Department of Immunology and Molecular Microbiology, Texas Tech University-Health Sciences Center, Lubbock, Texas, United States of America; 3 Department of Urology, Feinberg School of Medicine, Northwestern University, Chicago, Illinois, United States of America; China Medical University, TAIWAN

## Abstract

**Background:**

Although inflammation and prostate cancer (PCa) have been linked, the molecular interactions between macrophages and PCa cells are poorly explored. Pigment Epithelium-Derived Factor (PEDF) is an anti-angiogenic and anti-tumor factor. We previously showed that PEDF induces macrophages recruitment *in vitro*, correlates with macrophages density in human prostate, and stimulates macrophages polarization towards the classically activated pathway. Here, we demonstrate that PEDF modulates the interaction between macrophages and PCa cells through a bidirectional signalling leading to tumor cell apoptosis and phagocytosis.

**Methods:**

RAW 264.7 and THP-1 cells, and BMDMs were grown *in vitro* as mono- or co-cultures with PC3 or CL1 tumor cells. The effects of PEDF and its derived P18 peptide were measured on macrophages differentiation, migration, and superoxide production, and tumor cell apoptosis and phagocytosis. PEDF receptors (ATP5B, PNPLA2, and LRP6) and CD47 mRNA and protein expression were quantified in macrophages and tumor cells by quantitative RT-PCR, western blot, immunofluorescence and flow cytometry.

**Results:**

We found that PEDF induced the migration of macrophages towards tumor 3D spheroids and 2D cultures. In co-culture, PEDF increased PCa cells phagocytosis through an indirect apoptosis-dependent mechanism. Moreover, PEDF stimulated the production of superoxide by macrophages. Conditioned media from macrophages exposed to PEDF induced tumor cells apoptosis in contrast to control conditioned media suggesting that ROS may be involved in tumor cells apoptosis. ATP5B and PNPLA2 PEDF receptors on macrophages and CD47 on tumor cells were respectively up- and down-regulated by PEDF. As PEDF, blocking CD47 induced phagocytosis. Inhibiting ATP5B reduced phagocytosis. Inversely, PNPLA2 inhibition blocks differentiation but maintains phagocytosis. CD47-induced phagocytosis was partially reverted by ATP5B inhibition suggesting a complementary action. Similar effects were observed with P18 PEDF-derived peptide.

**Conclusions:**

These data established that modulating the molecular interactions between macrophages and PCa cells using PEDF may be a promising strategy for PCa treatment.

## Introduction

The tumor microenvironment is constituted by various cell types including inflammatory cells [1]. Among inflammatory cells, tumor-associated macrophages (TAMs) represent the predominant cell population. TAMs are characterized by a phenotypic plasticity and polarize into two main subsets, M_1_ (classically activated) or M_2_ (alternatively activated) macrophages depending on the surrounding environment. Accumulation of M_2_-type macrophages (Arg1+/IL12_Low_/ IL10_High_) or the enrichment of TAM-associated gene signatures have been correlated with poor prognosis and disease outcome in several types of cancer [2–5]. TAMs play a role in matrix remodeling and angiogenesis in multiple human tumors [6–8]. TAMs secreted a wide range of pro-angiogenic mediators such as bFGF, thymidine phosphorylase, uPA, and adrenomedullin [1]. At hypoxic tumor sites, HIF-1α up-regulates VEGF-A expression in TAMs [9] and, MMP-1, -7 and -9 proteolytic enzymes [10–12]. CXCL12 expression in gastric cancer and activation of the β-catenin pathway correlate with increased microvascular density and invasiveness [13, 14]. In the same cancer type, IL-25 was positively associated with histological grade and was found to be an independent predictor of favorable survival [15]. CCL18 and CXCL8 produced by TAMs were positively linked with microvessel density and metastatic potential in breast and thyroid papillary cancer [16, 17]. TAMs also produce immunosuppressive factors such as PGE_2_, IDO, TGFβ and IL10 to recruit immunosuppressive T regulatory cells [8]. TAMs serve as the main players for impeding the therapeutic activities of radiotherapy, chemotherapy, anti-hormonal, immunotherapy, and molecular targeting therapies [18–23] therefore emphasizing macrophages as an important therapeutic target.

In contrast to M_2_, M_1_ macrophages (iNOS+/IL12_High_/IL10_Low_) have tumoricidal activity, produce high amount of inflammatory cytokines, ROS and present a strong innate and adaptive immune activity. In non-small cell lung cancer, the M_1_, but not M_2_, macrophage density in tumor islets positively correlated with survival time [24]. Infiltration of diametrically polarized macrophages (M_1_/M_2_) predicts overall survival of patients with gastric and ovarian cancer, and renal cell carcinoma [25–27]. In PCa, Lissbrant et al. linked the volume density of TAMs to a shorter survival time, while Shimura et al. reported high TAMs number to be an independent predictor of disease-free survival after surgery for this disease [28, 29]. In agreement with Lissbrant’ study, the inhibition of macrophages function or pro-inflammatory pathways in PCa cells delayed tumor growth in experimental *in vivo* models [30]. Several TAM-targeting cancer therapy strategies are currently been tested: i) inhibiting macrophage recruitment; ii) suppressing TAM survival; iii) enhancing M_1_ tumoricidal activity of TAMs; and iv) blocking M_2_ tumor-promoting activity of TAMs [7]. While suppressing TAM recruitment/survival are attractive options [8], reprogramming toward an anti-tumor M_1_ phenotype appears to be a better target for clinical testing.

Pigment Epithelium-Derived Factor (PEDF) is a secreted angio-inhibitor with anti-tumor activities and suggested immune-modulatory properties [31–33]. We have previously demonstrated that PEDF induces the migration of macrophages and their polarization towards the classically activated pathway [34]. In human prostate, we showed that PEDF expression correlates with macrophage density. Accordingly, PEDF expression increased macrophages density in the orthotopic MatLyLu rat PCa model [35]. Still the precise role of PEDF in modulating the molecular interactions between macrophages and PCa cells remain uncharacterized. Herein, we report that PEDF directs macrophages towards tumor spheroids. Using co-culture, we showed that PEDF also stimulates the polarization of macrophages leading to tumor cell phagocytosis. PEDF-induced phagocytosis was apoptosis-dependent, but was not caused by a direct tumoricidal effect of PEDF on tumor cells. In contrast, PEDF stimulated the production of superoxide radicals by the macrophages. Furthermore, conditioned media from PEDF-treated macrophages induced apoptosis in tumor cells when compared to control media. ATP5B and PNPLA2, two known receptors for PEDF, were highly expressed in primary macrophages and cell line, and were up-regulated by PEDF. Inversely, the “don’t eat me” CD47 signal, expressed on the tumor cell surface was repressed by PEDF. Blocking CD47 induced phagocytosis. Inversely, inhibiting ATP5B significantly reduced phagocytosis. In contrast, inhibition of PNPLA2 blocks differentiation but maintains phagocytosis. PEDF’s inflammatory properties were reproduced by the PEDF-derived and anti-tumor P18 peptide. Thus, our data illustrated a novel PEDF-mediated signaling involving PNPLA2 up-regulation on macrophages to induce M_1_ polarization and, CD47 down-regulation on tumor cells which in collaboration with ATP5B elevation on macrophages leads to phagocytosis.

## Materials and methods

### Cells & reagents

The androgen-refractory PCa (CRPC) PC3, androgen-sensitive Tramp-C1, RAW 264.7 and THP-1 cells were from the ATCC (Manasses, VA). CRPC CL1 cell line was a gift from Dr. Belldegrun (Un. of California, LA, CA). CL1 cells were derived as an androgen-independent variant from the human androgen-sensitive LNCaP cells as described in [36]. PC3 were grown in RPMI1640 with 10% fetal bovine serum (FBS). PC3-DsRed Express (designated as PC3-Ctrl), PC3-DsRed Express-PEDF (designated as PC3-PEDF), CL1-DsRed express (CL1-Ctrl) and CL1-DsRed express-PEDF cells (CL1-PEDF, passages 4–12) were established and grown as we described in [36, 37]. Tramp-C1 cells were grown in DMEM with 5% nu serum/5% FBS, 0.005 mg/ml Bovine insulin and 10 nM DHT. RAW 264.7 and THP-1 cells were grown between passages 1–12 to block gene loss and impaired macrophage immune function using RPMI1640 with 10% FBS. For macrophages differentiation/activation, monocytes/macrophages were treated for 48 hrs with 50 nM PMA/phorbol 12-myristate 13-acetate (Sigma Aldrich, St. Louis, MO) followed by PEDF treatment (BioProducts, Middletown, MD) in serum-free medium. P18 was a gift from Dr. Volpert [38]. ZVAD was from BD Pharmingen (San Diego, CA). To inhibit PNPLA2, macrophages were treated for 30’ with 5 μM of the irreversible antagonist (S)-BEL (Cayman Chemical; Ann Arbor, MI) before PCa co-culture or with 40 μM Atglistatin (Sigma-Aldrich, Milwaukee, WI) at the time of the co-culture. For ATP5B inhibition, 10 nM angiostatin (BioVision; Milpitas, CA) was added to the co-culture. CD47 Ab was from BD Biosciences (B6H12, San Jose, CA).

### 3D tumor spheroid assays

Tumor spheroid assays were performed using the 3-D Culture Matrix™ Basement Membrane Extract Reduced Growth Factor (Trevigen, Gaithersburg, MD). 1x10^4^ CL1-Ctrl (Red) PCa cells/ml were diluted in 24ml of Assay Medium (98ml of growth media plus 2ml of 3-D Culture Matrix RGF BME). 500μl of PCa cell suspension were then added to each well of a 48 well plate pre-coated with 3-D Culture Matrix RGF BME. Cell growth and structure formation were observed every other day using the Nikon T1-E microscope. The medium was changed every three days. Once spheroids reached a diameter of ~600μm, 4x10^5^ of green-fluorescent macrophages (CellTracker Green CMFDA, Invitrogen) with or without PEDF or P18 peptide were added to each well. After 24 hours, macrophages migration towards the tumor spheroids was visualized using the Nikon T1-E 200 microscope (63X, Z-stack). Before imaging of the tumor spheroids within the 48-well plate, wells were filled with Cell Superfusion Buffer (CSB: 0.35mM Na2HPO4, 110mM NaCl, 0.44mM KH2PO4, 5.4mM KCl, 1mM MgSO4, 1.3mM CaCl3, 25mM HEPES, pH 7.4). After imaging, the Z-stack was merged, and the distance between the macrophages (Green) and the tumor spheroids (Red) was calculated using the two point analysis function (NIS-Elements AR 4.00.03, Carl Zeiss).

### Phagocytosis *in vitro* assay

RAW 264.7 and THP-1 macrophages were seeded (3.5–7.5x10^5^) on PCa CL1-Ctrl or PC3-Ctrl cells (2.5x10^4^; Red) in 6-well plates containing 25 mm round sterile cover glass in serum-free medium supplemented with 50 nM PMA ± 10 nM PEDF or P18 peptide [39]. Cells (100/condition treatment) were live imaged at 48 hours using the Nikon T1-E 200 microscope (63X, Z-stack). Before imaging, cover glass was placed in Attoflour^®^ Cell Chamber (Life Techn.) and filled with 1 ml of CSB. Region of interest (ROI) were selected, and the intensity surface plot function (NIS-Elements AR 4.00.03; S1 Fig) was used to measure the signal intensity of each ROI. ROI mean intensity was calculated from >30 representative ROI (S1 Fig). To perform spectral imaging microscopy and obtain a spectral output, a Spectra-Pro-300i spectrograph (Acton Research Instruments, Acton, MA) was directly coupled to the side port of an Olympus IX70 inverted microscope through a C-mount adaptor (IX-TVAD). We also used a high dynamic range from transfer back illuminated cryogenically cooled Charged Coupled Device (CCD) camera (Spec10:400 B, LN; 16bit, Princeton Instruments, Trenton, NJ). The CCD temperature was maintained at -100°C for all the experiments. The slit spectrograph was set at 200 μm throughout the experiments except for the zero order spectra, which was set to 3 mm. The length of the cell area was delimited by the 0.5 μm slit width and was divided into 16 discrete cellular regions of interest (S2 Fig, ROI were chosen in different areas around the cytoplasm of the cancer cells, the vacuoles of the macrophages that displayed red fluorescence, and various regions of the background to establish a baseline), allowing for images with high spectral, temporal, and spatial resolution. As negative controls, ROIs were selected in non-fluorescent cytoplasmic and extra-cellular regions of RAW 264.7 cells grown as mono- or co-culture. The optical filters used for the excitation of dsRed-Express were 545 nm narrow bandpass excitation filter, 570 nm long bandpass dichroic mirror. A 60x objective (N.A 1.4; oil) was used. The fluorescence data was converted to ASCII format, prior to analysis with SigmaPlot (version 8.0).

### Cell cycle analysis

CL1 PCa cells were initially seeded in a 6-well dish at 1 X 10^5^ cells/well and allowed to grow for 48 hours before being treated with or without 10 nM PEDF. Cells were harvested with trypsin and fixed overnight in 70% ethanol at 4°C. Cells were then stained with a solution of 69 μM propidium iodide in 38 mM Sodium Citrate (pH 7.4), and incubated for 45 minutes at 37°C. For the cell cycle analysis, cells were initially gated for singlet cells before recording 10,000 events using a BD FACS Aria (BD Biosciences). Within each experiment, each sample was tested in triplicate; each experiment was repeated at least three times.

### TUNEL labeling

RAW 264.7 macrophages were incubated in serum-free medium with or without 10 nM PEDF. After 48 hours, cell conditioned Media (CM and CM-PEDF) were collected and cleared from cell debris by centrifugation. The supernatant was then added to 70% confluent CL1 cells. After 24 hours, tumor cells were fixed and stained with ApopTag kit (Millipore, Norcross, GA). Apoptosis was measured in >200 cells per treatment using the Zeiss Axiovert microscope as we previously described in [33]. Nuclei were counter-stained with 4’, 6-diamino-phenol-indole (DAPI).

### Cellular ROS/superoxide radicals

Free radicals and other reactive species were detected using the Cellular ROS/Superoxide Detection Assay Kit (Abcam) as recommended by the manufacturer. 1x10^4^ RAW 264.7 macrophages per 96-well were seeded and treated as for the phagocytosis assay. After 48 hours, the culture medium was removed. The cells were then incubated with the ROS/Superoxide Detection Mix for 30 min at 37°C. After washing, Oxidative Stress (ROS; Green) and O2^-^ (Red) -positive cells were counted and reported to the total amount of cell using the Zeiss Axiovert microscope. A minimum of 200 cells were counted per treatment condition and each treatment was tested in quadruplicate within a single experiment.

### Bone Marrow-Derived Macrophages (BMDMs) collection

BMDMs were collected from C57BL6 tumor-free and tumor-bearing mice. For the tumor-bearing mice, 5x10^6^ Tramp-C1 cells were s.c. injected into the hindquarters of male C57BL6 mice (6–8 weeks, Charles River). After 3 weeks, BMDMs were collected from the mice femur and tibia [40]. Cells were seeded at low density in RPMI1640 plus 10% FBS and 10% L929 cell condition media. Experiments were conducted after a minimum of ten days. Purity (> 95%) was verified by flow cytometry on a BD FACS Aria (BD Biosciences; 10,000 events counted) using F4/80 and Mac1 macrophages-specific markers (BD Pharmingen). This study was carried out in strict accordance with the recommendations in the Guide for the Care and Use of Laboratory Animals of the National Institutes of Health. The protocol was approved by Texas Tech University Health Sciences Center Institutional Animal Care and Use Committee (A3056-1).

### Immunofluorescence

60% confluent RAW 264.6 macrophages and BMDMs were fixed with 4% paraformaldehyde, quenched with 50mM PBS-NH_4_Cl, and blocked with 1% PBS-BSA. Cells were then incubated with α-ATP5B (Santa Cruz Biotechn., Dallas, TX) or α-PNPLA2 (R&D Systems, Minneapolis, MN) and incubated with fluorescent tag secondary antibody. Cells were mounted to cover slide with ProLong^®^ Gold antifade (LifeTechnologies, Grand Island, NY) reagent containing DAPI nuclei staining. ~200 cells/condition were imaged using the Zeiss AxioVert 200 microscope. CD47 and LAMP1 were visualized on living cells. CD47-blocking antibody (100 ng/μl, B6H12) was added to the tumor cells 30 minutes before co-culturing with macrophages. α-LAMP1 (100 ng/μl, 1D4B, BD Transduction Lab) was added at the time of the co-culture.

### RNA extraction and qRT-PCR

RNAs were extracted (RNeasy kit. Qiagen, Valencia, CA) and converted into cDNA (Verso cDNA kit, Thermo Scientific, Pittsburgh, PA). PEDF receptors cDNA: ATP5B (5′-GTTCATCCTGCCAGAGAC-3′/5′-CGATGACTGCCACGATT-3′), PNPLA2 (5′-TCCAAGGGGTGCGCTAT-3′/5′-GCTCATAAAG TGGCAAGTTGTC-3′), LRP6 (5′-TCTCCGGCGAATTGAAAG-3′/5′-GAGTCTTCTAGCACGATCC TGT-3′; Thermo Scientific), CD47 (NM_001777.3), and SIRPα (NM_007547.4, Qiagen) were amplified: 1× heating for 10′ at 95°C; 40× denaturating for 15″ at 95°C, followed by annealing/extension 1′ at 60°C/60× extension for 20″ at 60°C. As reference, S15 (5’-CAACGGCAAGA CCTTCAAC-3’/5’-GGCTTGTAGGTGATGGAGAAC-3’) was amplified; *C*_T_ values were determined by automated threshold analysis with MyIQ version 1.0 software. Fold change for each gene was calculated using the ΔΔ*C*_t_ method. Each sample was tested in triplicate. For the CD47 and SIRPα qRT-PCR, RNAs were extracted directly from the PCa-macrophages co-culture, based on the fact that CD47 and SIRPα are exclusively expressed by PCa and macrophage cells, respectively [41].

### Western blot

Whole cell lysates (50 mM Tris, 5 mM EDTA, 150 mM NaCl, 0.5% NP-40, 1 mM PMSF, pH 8.0) were separated by 4–15% SDS-PAGE and transferred onto PVDF membrane. After blocking (10 ml Tris-base, 100 ml NaCl, 0.1% Tween 20 (pH 7.5) with 5% nonfat dry milk), membranes were exposed to α-ATP5B or PNPLA2, and horseradish peroxidase-conjugated secondary Ab and; developed (ECL kit, Amersham, Pittsburgh, PA). Protein loading was controlled using β-actin.

### Plasma membrane CD47 staining

CL1-Ctrl and CL1-PEDF PCa cells (1x10^6^) were incubated with α-CD47/isotype FITC (B6H12, 200 ng/μl), and analyzed using the INSPIRE ImageStreamX MKII Amnis (3x10^4^ events). Plasma membrane CD47 was quantified using the masking function of the IDEAS Analysis v6.1software.

### Statistical analyses

Data are expressed as mean ± standard deviation of two to three independent experiments, each done at least in triplicate. Statistical evaluation of the data was done using Student’s T-test or One-Way analysis of variance (ANOVA) where appropriate (SPSS 23 software for Windows). P values < 0.05 (*) were considered statistically significant.

## Results

### PEDF stimulates the migration towards tumor spheroids and the phagocytic activity of macrophages *in vitro*

**Fig 1:**
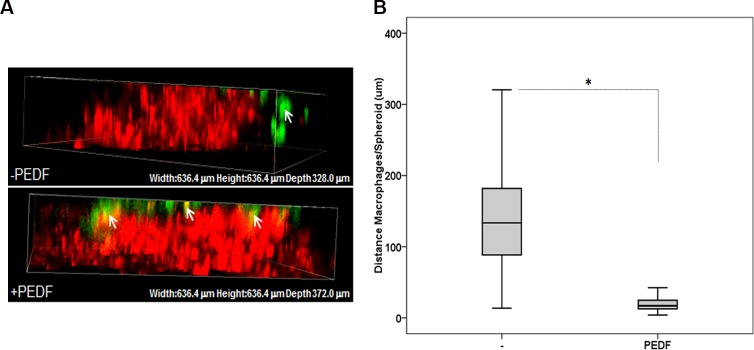
PEDF induces the migration of RAW 264.7 macrophages toward 3D CL1 tumor spheroids *in vitro*. (**A**) Migration of activated RAW 264.7 macrophages (Green) assessed by confocal microscopy, towards 3D CL1-Ctrl tumor spheroids (Red) ± 10 nM PEDF for 24 hours. (**B**) Quantitative analyses using the two point analysis function (NIS-Elements AR 4.00.03, Carl Zeiss) and showing that PEDF stimulates significantly the migration of macrophages towards the spheroids. Data points represent mean ± SD of triplicate samples from three independent experiments (*p < 0.05). A minimum of 30 measurements per treatment condition were taken within a single experiment. Statistical analyses were performed using the Student's t test.

We have previously demonstrated that PEDF induces the migration and polarization of human and mouse macrophages towards a M_1_ phenotype *in vitro*. We have also shown that PEDF expression positively correlates with macrophage density in the human prostate gland [34]. To link PEDF inflammatory activity to PCa we now investigate the effect of PEDF on the migration of macrophages towards prostate tumor spheroids and the phagocytosis of PCa cells *in vitro*. Using the highly reproducible CL1 tumor spheroids model, we demonstrated that macrophages (Green) were located in the periphery of the control spheroids (Red). In contrast, in the presence of PEDF, macrophages were found on the surface or infiltrating the spheroids (Fig 1A; arrows). Using the two point analysis function of the NIS-Elements AR 4.00.03 software (Carl Zeiss), we measured the distance between the spheroid and macrophages, and confirmed that PEDF directs the macrophages towards the spheroid (Fig 1B). To note, PEDF had no apparent effect on the tumor cell morphology within the spheroids. Similarly to the tumor spheroid, in the inverted Boyden chamber assay, the migration of RAW 264.7 macrophages was specifically induced by PEDF endogenously expressed by PC3 and CL1 PCa cell lines (S3 Fig).

**Fig 2:**
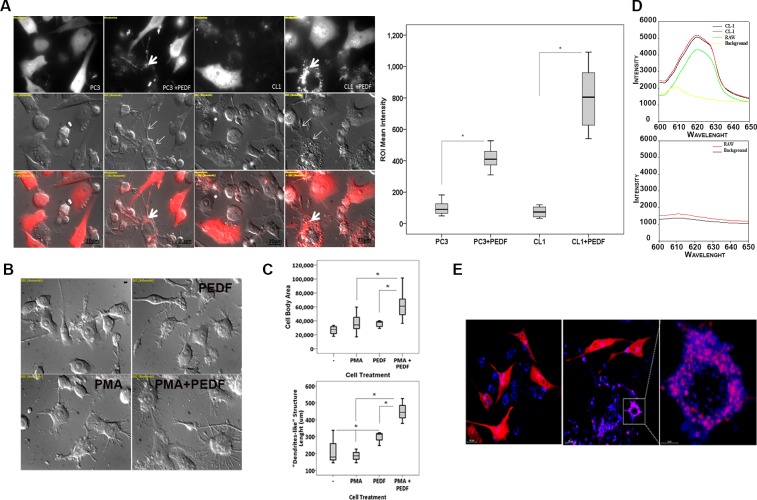
PEDF increases the phagocytosis of PCa cells by RAW 264.7 macrophages. (**A, Left**) Representative fluorescent and Nomarski images of PC3-Ctrl or CL1-Ctrl PCa cells (Red) co-cultured with RAW 264.7 macrophages ± PEDF (10 nM). Thin arrows denote macrophages differentiation. Thick arrows show tumor cell debris (Red) within the macrophages. Nomarski and confocal images were obtained using the Nikon T1-E microscope with A1 confocal and STORM super-resolution with a 63x objective (N.A. 1.4; oil). After imaging, Regions of Interest (ROIs) were selected, and the intensity surface plot function (NIS-Elements AR 4.00.03) was used to measure the signal intensity of each ROI. ROI mean intensity was calculated from >30 representative ROI. **Right:** Quantification of tumor cell phagocytosis. Data points represent ROI mean intensity ± SD of triplicate samples per treatment condition from three independent experiments. Data were represented using a boxplot graph (SPSS 23 software for Windows) showing the median, inter-quartile range, upper and lower quartiles, and whiskers. Statistical analysis was performed using the Student's t test. *: P<0.05. (**B**) Representative Nomarski pictures of RAW 264.7 mono-culture treated for 48 hours with PMA (50 nM), PEDF (10 nM), control, or PMA/PEDF supportive of significant changes in macrophages cell morphology (cell body size and dendrite length) in the presence of PEDF with or without PMA. (**C**) Quantification of body sizes and dendrite length in RAW 264.7 macrophages measured using ImageJ and showing that PEDF significantly increased the body size and dendrite length of macrophages when compared to untreated control. Data points represent mean ± SD of triplicate samples from three independent experiments. Statistical analysis was performed using ANOVA followed by the Tuckey test, *: p <0.05. (**D**) Spectral imaging microscopy showing the same wavelength (DsRed Express) in the engulfment vesicles than in the cytoplasm of CL1-Ctrl tumor cells. In contrast, no fluorescence was detected within the cytoplasm of RAW 264.7 cultured in the absence of PCa cells and in the extra-cellular space of the co- and mono-cultures. (**E**) LAMP1 (blue) immuonostaining in CL1 (Red)–RAW 264.7 co-cultures treated ± PEDF denoting that LAMP-1 co-localize (Pink) with tumor cell debris (Red) within RAW 264.7 macrophages.

**Fig 3:**
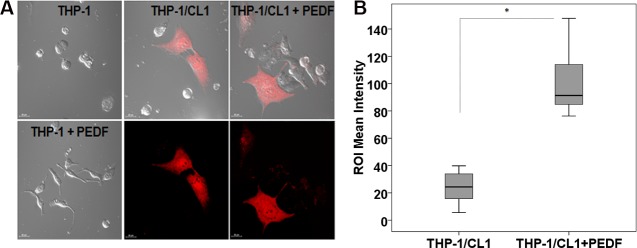
Validation of PEDF stimulatory effect on the phagocytosis of PCa cells using human THP-1—CL1 co-culture. **(A**) Representative fluorescent and Nomarski pictures of THP-1 macrophages grown as mono- or co-culture with CL1-Ctrl PCa cells (Red) ± PEDF (10 nM). Nomarski and confocal images were obtained using the Nikon T1-E microscope with A1 confocal and STORM super-resolution with a 60x objective (N.A. 1.4; oil). After imaging, Regions of Interest (ROIs) were selected, and the intensity surface plot function (NIS-Elements AR 4.00.03) was used to measure the signal intensity of each ROI. ROI mean intensity was calculated from >30 representative ROI. **(B)** Quantification of tumor cell phagocytosis (ROI mean intensity) averaged from three different experiments. Data were represented using a boxplot graph showing the median, inter-quartile range, upper and lower quartiles, and whiskers. Statistical analysis was performed using the Student's t test. *: P<0.05.

To evaluate PCa cells phagocytosis, we performed cytotoxicity assays by co-culturing mouse RAW 264.7 macrophages and target human PC3 and CL1 PCa cells under PEDF/control treatments as described in [42]. As expected, PEDF induced the differentiation of macrophages when compared to untreated control (Fig 2A; thin arrows). In contrast, no apparent effects on tumor cell morphology were observed. PEDF differentiation effect on macrophages was direct as demonstrated by the significant increase in body size and dendrite length (Fig 2B and 2C) concomitant to a M1 polarization profile macrophages [34] in macrophage mono-culture. In the co-culture, PEDF also stimulated the engulfment of PC3 and CL1 cell lines by the macrophages by a factor of 4 and 8 times, respectively (Fig 2A; thick arrows). The spectral analysis of the red fluorescence present in the macrophages endocytosis vesicles was identical to the one found in the cytoplasm of tumor cells confirming that tumor cells debris are internalized by macrophages (Fig 2D). In contrast, no fluorescence was detected within the cytoplasm of RAW 264.7 mono-culture and in the extra-cellular space of co- and mono-cultures. To validate phagocytosis, we used LAMP-1 (Lysosomal Associated Membrane proteins are required for fusion of lysosomes with phagosome, later phagosomal stages and phagosomes after phagosomal fusion [43, 44]) as a specific marker of the late stage of phagocytosis. In this experiment, we demonstrated that tumor cell debris (Red) co-localized with LAMP1 (Blue) within the macrophage vesicles corroborating their phagosome-related identity (Fig 2E). Similar to RAW 264.7 cells, we found that PEDF increased by 5 fold the phagocytosis of CL1 cells by human THP-1 macrophages (Fig 3).

### PEDF stimulatory effect on tumor cells phagocytosis is apoptosis-dependent and concomitant to the production of superoxide by macrophages

**Fig 4:**
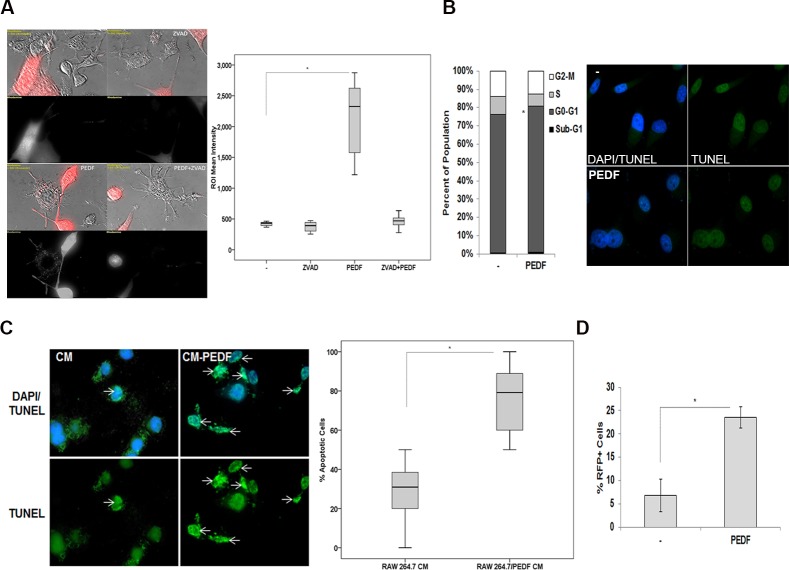
PEDF induces the phagocytosis of tumor cells through an apoptosis-dependent mechanism and possibly via the production of Superoxide radicals. (**A, Left**) Representative images of CL1-Ctrl PCa cells (Red) and RAW 264.7 macrophages treated with buffer control, PEDF (10 nM), ZVAD (40 μM), or combination. (**A, Right**) Quantification of tumor cell phagocytosis (ROI mean intensity) averaged from two different experiments. Data were represented using a boxplot graph showing the median, inter-quartile range, upper and lower quartiles, and whiskers. Statistical analysis was performed using the Student's t test. *: P<0.05. (**B, Left**) Cell cycle distribution of CL1 cells exposed to PEDF (10 nM). Data points represent mean ± SD of triplicate samples from two independent experiments. Error bars denote ± SD (*p < 0.05). (**B, Right**) Representative images of fragmented DNA (Green) in CL1 cells treated with PEDF (10 nM) or control showing that PEDF has no direct tumoricidal effect on tumor cells. Nuclei were visualized by DAPI (blue) staining. (**C, Left**) Representative images of fragmented DNA (Green) in CL1 cells treated with RAW 264.7 CM or CM-PEDF. Nuclei were visualized by DAPI (blue) staining. (**C, Right**) Apoptosis quantification measured by counting the number of apoptotic cells (Green) over the total amount of cells (>100 cells). Data points represent mean ± SD of triplicate samples from two independent experiments. Error bars denote ± SD (*p < 0.05). (**D**) Quantification of Superoxide production by RAW 264.7 macrophages treated ± PEDF (10nM). Data points represent mean ± SD of quadruplicate samples from two independent experiments; *: p < 0.05.

To identify the underlying mechanisms resulting in PCa cell phagocytosis under PEDF treatment, we used the caspase broad inhibitor ZVAD-FMK. Our data clearly demonstrated that while ZVAD-FMK alone had no effect on phagocytosis, it abolished PEDF-induced phagocytosis (Fig 4A) implying an apoptosis-dependent mechanism. To investigate a possible direct tumoricidal effect of PEDF on tumor cells, we analyzed the cell cycle distribution of CL1 cells treated with or without PEDF. Interestingly, cell death remained unchanged by PEDF suggesting a macrophages-driven action (Fig 4B; Left panel). Accordingly, no apoptosis was detected in CL1 cells treated with PEDF or control (Fig 4B; Right panel). In contrast, the G1 population increased in response to PEDF corroborating PEDF effect on PCa cells differentiation as we previously demonstrated in [45]. We then treated CL1 cells with media conditioned (CM) by RAW 264.7 cells exposed to PEDF or buffer control. While % apoptosis was low in untreated CL1, % apoptosis was found significantly elevated in CM-PEDF (Fig 4C). In the meantime, PEDF treatment increased the amount of superoxide produced by the macrophages suggesting that superoxide production could cause tumor cell apoptosis (Fig 4D; S4 Fig). In contrast, no significant increase in ROS production by macrophages could be found (data not shown). Our data demonstrate therefore that PEDF induces the migration of macrophages towards tumors and that, by acting on macrophages; PEDF stimulates their polarization into M1 macrophages resulting subsequently into superoxide production, tumor cell apoptosis and phagocytosis.

### P18 mimics PEDF inflammatory effect *in vitro*

**Fig 5:**
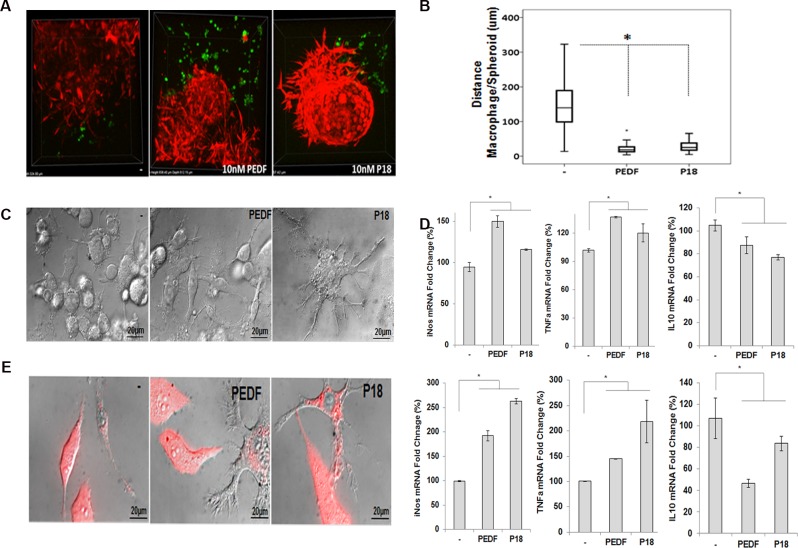
The synthetic P18 peptide mimics the inflammation-related function of PEDF. (**A**) Migration of macrophages (Green) assessed by confocal microscopy, towards 3D CL1 spheroids (Red) treated with PEDF (10 nM), P18 peptide (10 nM) or control buffer for 24 hours. (**B**) Quantitative analyses using the two point analysis function (NIS-Elements AR 4.00.03, Carl Zeiss) and showing that as PEDF, P18 peptide significantly stimulates the migration of macrophages towards the spheroids. Error bars show SD of the mean, based on two independent experiments. Statistical analyses were performed using the Student’s t test, *: p < 0.05. (**C**) Nomarski representative images of RAW 264.7 cells treated with PEDF (10 nM), P18 peptide (10 nM) or control buffer indicative of significant changes in macrophages morphology under PEDF and P18 exposure (10 nM). (**D**) Total RNAs from RAW 264.7 (Top graphs) and BMDMs (tumor-bearing mice; bottom graphs) treated with or without PEDF and P18 were analyzed by qRT-PCR for iNOS, TNFa, and IL10 mRNAs. Results were normalized to S15 and are presented as relative fold change compared to expression levels in non-treated cells. Data points represent mean ± SD of triplicate samples from two independent experiments. Statistical analyses were performed using the Student’s t test, *: p < 0.05. (**E**) Representative Nomarski/confocal images of CL1-Ctrl PCa cells (Red)–macrophages co-cultures demonstrating that as PEDF, the P18 peptide (10 nM) induced the phagocytosis of CL1 cells *in vitro*.

The P18 peptide (residues 39–57 of the whole PEDF) has been identified as PEDF anti-angiogenic and anti-tumoral functional epitope [33, 38]. To determine if this epitope could be involved in PEDF inflammatory role, P18 activity was tested *in vitro*. As PEDF, P18 stimulated significantly the migration of macrophages towards tumor spheroids (Fig 5A and 5B). P18 also modified the cell morphology of macrophages *in vitro* (Fig 5C). Phenotypic results were corroborated by increased iNOS and TNFa, and decreased IL10 in both RAW 264.7 macrophages and BMDMs treated with PEDF or P18 (Fig 5D). In agreement with a M_1_ polarization, P18 was also able to induce the phagocytosis of PCa cells *in vitro* (Fig 5E).

### PEDF induces the expression of ATP5B and PNPLA2 in macrophages

**Fig 6:**
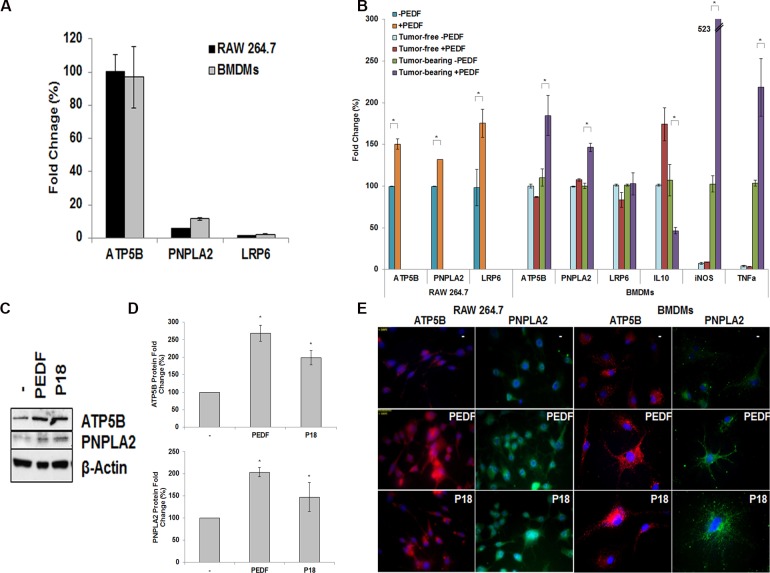
PEDF receptors expression *in vitro*. (**A**) Total RNAs from RAW 264.7 and BMDMs (tumor-free mice) were analyzed by qRT-PCR and normalized to S15. Results are presented as relative fold change compared to ATP5B mRNA expression levels in macrophages. Data points represent mean ± SD of triplicate samples from two independent experiments. Statistical analyses were performed using the Student’s t test, *: p < 0.05. (**B**) Expression levels of ATP5B, PNPLA2, and LRP6 mRNAs in RAW 264.7 cells and BMDMs collected from tumor-free and tumor-bearing mice, quantified by qRT-PCR and normalized to S15 mRNA. Data points represent mean ± SD of triplicate samples from two independent experiments. Error bars denote ± SD (*p < 0.05). (**C**) Western blot showing the expression levels of ATP5B and PNPLA2 in RAW 264.7 cells treated with PEDF (10 nM), P18 (10 nM) or control. Actin is used as a loading control. (**D**) Quantification of ATP5B and PNPLA2 protein levels averaged from three different experiments. Error bars show standard error of the mean. *: P <0.05. (**E**) ATP5B (Red) and PNPLA2 (Green) immunostaining of RAW 264.7 macrophages and BMDMs collected from tumor-bearing mice. Macrophages were treated with PEDF (10 nM), P18 (10 nM) or buffer control. Nuclei are visualized by DAPI (Blue) staining.

To characterize the molecular effectors involved in PEDF inflammatory effect, we measured the expression levels of three of the known PEDF receptors (ATP5B, PNPLA2, LRP6) in macrophages. ATP5B and PNPLA2 were found expressed in a greater extent than LRP6 in RAW 264.7 cells and BMDMs (Fig 6A). Although PEDF significantly stimulated the expression of all three receptors in RAW 264.7 cells, only ATP5B and PNPLA2 were up-regulated in BMDMs by PEDF (Fig 6B). Furthermore, this increase was only found in BMDMs collected from tumor-bearing mice, but not from tumor-free mice [46] emphasizing the need of tumor sensitization/activation as seen in tumor angiogenesis [31]. Interestingly, BMBMs collected from tumor-bearing mice demonstrated a M1 phenotype after PEDF treatment suggesting again a link between differentiation, and PEDF and PEDF receptors expression levels. In agreement with the qPCR results, PEDF stimulates by 2.7 and 2 fold ATP5B and PNPLA2 protein levels, respectively (Fig 6C and 6D). As well, immunofluorescence studies demonstrated elevated ATP5B and PNPLA2 in RAW 264.7 and BMDMs exposed to PEDF suggesting the involvement of both ATP5B and PNPLA2 in PEDF modulating properties on macrophages (Fig 6E). Both receptors were also induced by P18 peptide therefore confirming that P18 mimics the modulatory action of PEDF on inflammation (Fig 6C, 6D and 6E).

### PEDF represses CD47 expression in PCa cells

**Fig 7:**
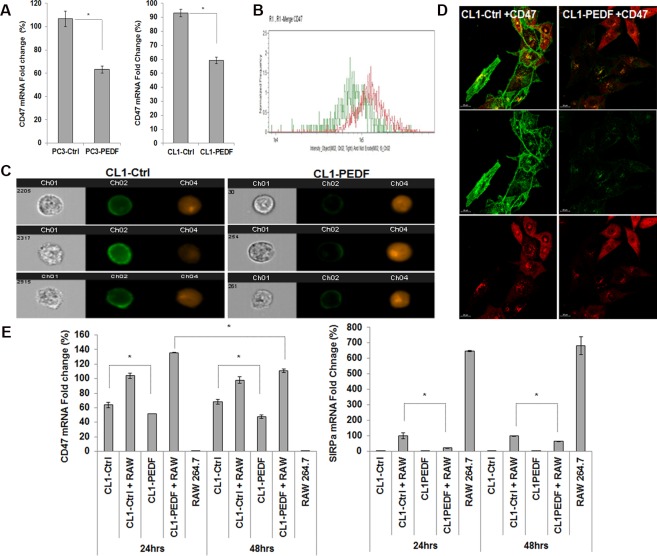
PEDF down-regulates CD47 *in vitro*. (**A**) Total RNAs from PC3-Ctrl, PC3-PEDF, CL1-Ctrl, and CL1-PEDF were analyzed by qRT-PCR and normalized to S15. Results are presented as relative fold change compared to control cells. Data points represent mean ± SD of triplicate samples from two independent experiments. Statistical analyses were performed using the Student’s t test, *: p < 0.05. (**B**) Plot, obtained by flow cytometry (30,000 events) showing the reduced plasma membrane expression of CD47 in CL1-PEDF (Green) compared to CL1-Ctrl cells (Red). (**C**) Set of three representative pictures of CL1-Ctrl (**Left**) and CL1-PEDF cells (**Right**) with ch1: Brightfield; ch2: CD47; and ch4: DsRed Express (tumor cells). (**D**) CD47 staining (Green), assessed by confocal microscopy in CL1-Ctrl and CL1-PEDF (Red). Representative pictures from three different experiments. (**E**) Total RNAs from CL1-Ctrl, CL1-PEDF, and RAW 264.7 mono-cultures, and PCa/RAW 264.7 co-cultures were analyzed by qRT-PCR and normalized to S15. Results are presented as relative fold change compared to control CL1-Ctrl/RAW 264.7 co-cultures. Data points represent mean ± SD of triplicate samples from two independent experiments. Statistical analyses were performed using ANOVA followed by the Tukey test, p < 0.05.

Tumor immune evasion through phagocytosis inhibition is required for tumor progression [47]. Cell surface expression of CD47 (also known as integrin-associated protein) is a common mechanism by which cells protect themselves from phagocytosis [41, 48]. CD47 is a widely expressed trans-membrane protein, and has been showed to be markedly increased in solid tumors including PCa when compared with their normal counterparts [41, 48]. CD47 have been involved in multiple cellular functions including apoptosis, proliferation, adhesion and migration with both beneficial and detrimental effects depending on the cell types and the binding partners involved [41]. Another function is to serve as a ligand for the signal regulatory protein-α (SIRPα), a protein expressed on macrophages and dendritic cells [49]. Upon binding to CD47, SIRPα initiates a “don’t eat me” signaling cascade that results in the inhibition of phagocytosis [50]. To determine if CD47 could be involved in PEDF-stimulated phagocytosis, we measured CD47 expression in PC3 and CL1 tumor cells expressing PEDF or control plasmid. In both cell lines tested, PEDF expression was concomitant to a 30–40% inhibition in CD47 mRNA (Fig 7A). Accordingly, CD47 localization at the plasma membrane was significantly decreased by PEDF (Fig 7B, 7C and 7D) validating CD47 as a target of PEDF. In co-cultures, although CD47 remained unchanged under PEDF treatment, CD47 expression was reduced over time by PEDF suggesting that in PCa-macrophages co-cultures in which CD47 mRNA expression is increased, PEDF may need more time to repress CD47 mRNA or that macrophages could produce secreted or surface factor(s) inhibiting PEDF effect on CD47 (Fig 7E). Accordingly, SIRPα expression was repressed by PEDF emphasizing that PEDF could disturb CD47-SIRPα interaction causing phagocytosis (Fig 7E).

### Role of CD47, ATP5B and PNPLA2 in PEDF’s inflammatory effect

**Fig 8:**
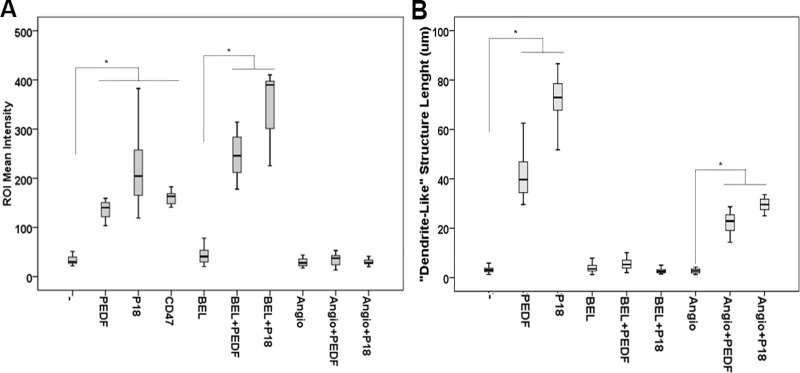
Role of PNPLA2, ATP5B and CD47 in macrophages differentiation and phagocytic activity. (**A**) Quantification analyses of PCa cell phagocytosis in CL1-Ctrl/RAW 264.7 co-cultures treated with α-CD47 (100 ng/μl), PEDF or P18 (10 nM) alone or PEDF/P18 in combination with either S-BEL (5 μM) or Angiostatin (10 nM). Data points represent ROI mean intensity ± SD of triplicate samples per treatment condition from two independent experiments. (**B**) Quantification analyses of the «dendrite-like» structure length using the ImageJ software. Data points show the mean ± SD of triplicate samples per treatment condition from two independent experiments. Statistical analyses were performed using the Welch and Brown-Forsythe tests followed by the Games-Howell test, *: p < 0.05.

**Fig 9:**
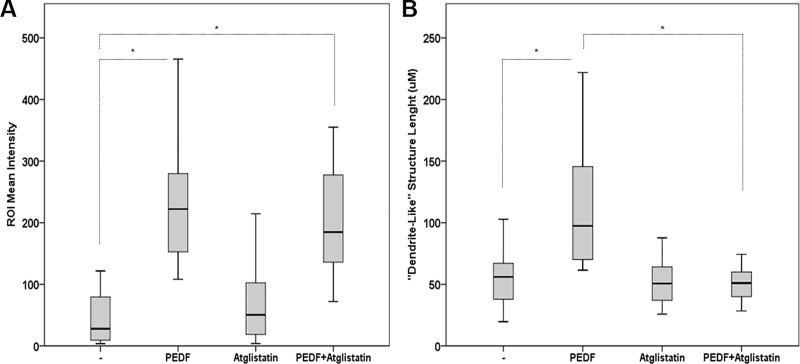
Effect of Atglistatin on macrophages differentiation and phagocytic activity. (**A**) Quantification analyses of PCa cell phagocytosis in CL1-Ctrl/RAW 264.7 co-cultures treated with PEDF (10 nM), Atglistatin (40 μM) or PEDF/Atglistatin combination. Data points represent ROI mean intensity ± SD of triplicate samples per treatment condition from two independent experiments. (**B**) Quantification analyses of the «dendrite-like» structure length using the ImageJ software. Data points show the mean ± SD of triplicate samples per treatment condition from two independent experiments. Statistical analyses were performed using the Welch and Brown-Forsythe tests followed by the Games-Howell test, *: p < 0.05.

**Fig 10:**
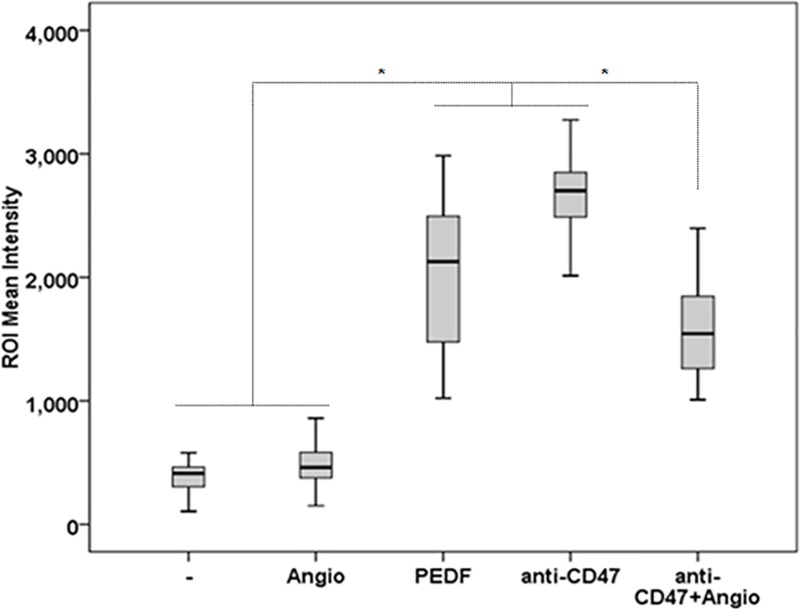
Effect of α-CD47 and Angiostatin combination on the phagocytosis of CL1 cells. Quantification analyses of PCa cell phagocytosis in CL1-Ctrl/RAW 264.7 co-cultures treated with α-CD47 (100 ng/μl) or Angiostatin (10 nM) alone or in combination. Data points represent ROI mean intensity ± SD of triplicate samples per treatment condition from two independent experiments. Statistical analyses were performed using the Welch and Brown-Forsythe tests followed by the Games-Howell test, *: p < 0.05.

Our findings suggest that PEDF and the P18 peptide modulate the molecular interactions between PCa cells and macrophages through bidirectional signaling. While a *cis signal* would result in the down-regulation of CD47 in PCa cells leading to tumor cell phagocytosis, a *trans signal* could be initiated on the macrophage cell surface leading subsequently to PNPLA2 and ATP5B elevation, and induction of macrophage differentiation and phagocytic activity, respectively. To test this hypothesis, we used CD47 blocking antibody to induce phagocytosis [51, 52]. In parallel, we used (S)-BEL to inhibit the calcium-independent PNPLA2, and Angiostatin as a binding competitor of PEDF on ATP5B [53, 54]. After treatment, we then quantified macrophages differentiation and phagocytic activity. Similar to PEDF and P18, CD47 blocking antibody stimulated phagocytosis (Fig 8A, S5 Fig). While PEDF and P18 induced the differentiation and phagocytic activity in macrophages, (S)-BEL co-treatment decreased significantly macrophages differentiation while phagocytosis remained unchanged (Fig 8A and 8B). Because S-BEL has been described as an inhibitor for both PNPLA2 [iPLA2ζ, [55]] and PNPLA9 (iPLA2β), we reproduced the experiment using Atglistatin, the first selective inhibitor for PNPLA2. As expected, our data showed that both S-BEL and Atglistatin treatments inhibited macrophage differentiation without affecting phagocytosis therefore validating PNPLA2 role in tumor cell differentiation (Fig 9). Inversely, angiostatin completely inhibited PEDF phagocytic activity. Angiostatin inhibitory effect was not due to other of its known receptors (Annexin 2, c-Met, integrin α_V_β_3;_ S6 Fig). Surprisingly, Angiostatin treatment also resulted in a slight decrease in dendrite-length, an effect we think could be explained by Angiostatin disrupting effect on monocyte/macrophage actin cytoskeleton [56]. While the combination of PNPLA2 inhibitors and Angiostatin was toxic and did not allow us to analyze their combined effects on macrophage differentiation and phagocytosis, we investigated the relative role of CD47 and ATP5B in tumor cell phagocytosis. For this purpose, the CD47-blocking antibody was tested with angiostatin. Our data showed that angiostatin partially reversed phagocytosis-induced by CD47 blocking (Fig 10) suggesting that both CD47 and ATP5B may participate into phagocytosis.

## Discussion

Compelling evidence indicated that TAMs are playing a crucial role in cancer from the initiation step to tumor growth and metastasis. However, current published data using TAMs quantification in PCa remain contradictory [28, 29, 57–59] suggesting that the molecular interactions between TAMs and other cell populations from the tumor microenvironment need to be also investigated to establish better predictive tools. While PEDF’s role in inflammation-related diseases have been widely described [60, 61], our study represents the first demonstration that PEDF can modulate the interactions between PCa cells and macrophages at a molecular level. In our model, we propose that PEDF may act through a bidirectional signaling to induce apoptosis and phagocytosis of prostate tumor cells. While the *cis signal*, located in tumor cells, would lead to the repression of CD47 by PEDF and initiation of tumor cell phagocytosis, the *trans signal* would participate in the polarization and, tumoricidal and phagocytic activities of macrophages through the PNPLA2 and ATP5B initiated pathways, respectively. PNPLA2 (ATGL) was the first putative receptor identified for PEDF [55]. While highly expressed in adipose tissue, ATGL is also expressed in less extent in cardiac and skeletal muscles as well as in testis tissue [62]. Furthermore, ATGL is expressed in macrophages and, in agreement with our data, ATGL-/- macrophages showed a defect in cell polarization and migration [63]. Accordingly, treatment with S-BEL and Atglistatin reversed PEDF-induced macrophages differentiation but maintained tumor cell phagocytosis. Concomitantly, we have shown that S-BEL inhibits PNPLA2 protein expression but not ATP5B (data not shown) again suggesting a link between PEDF-induced PNPLA2 up-regulation and macrophages differentiation. In contrast, the membrane protein F1 ATP synthase is localized on endothelial cells and tumor cells [64] and has been linked to the anti-angiogenic functions of PEDF [54, 65]. The involvement of CD47 represents an important finding as blocking CD47-SIRPα interaction is currently being investigated as a therapeutic target in clinical trials [51, 66, 67]. Also, while the prognostic significance of CD47 in PCa is unknown, our data suggest that CD47 may be a good target for this type of cancer. The relative role of CD47 down-regulation and ATP5B up-regulation in phagocytosis is currently under investigation. Our preliminary data suggest that both CD47 and ATP5B may be involved (Fig 10). One could imagine that while CD47 repression triggers phagocytosis, ATP5B may play an instrumental role in the acidification of the phagocytic vesicles. Accordingly, we demonstrated that blocking PEDF binding to ATP5B using Angiostatin [54] significantly reduced phagocytosis. While we are aware that angiostatin binds to other cell surface receptors (Annexin 2, c-Met and integrin α_V_β_3_)_,_ we do not believe these receptors to be involved based on our findings summarized in S6 Fig. While Annexin 2 mRNA is slightly repressed (18%) by PEDF, Annexin 2 expression has been conversely associated with increased phagocytosis [68]. Although c-Met mRNA levels varied with PEDF and angiostatin treatment, the c-Met signaling has been shown not to have an effect on modulating cytokine expression, phagocytosis, or antigen presentation but instead promotes proliferation of macrophages [69]. As well, while the integrin α_V_β_3_ has been described as necessary for phagocytosis, their inhibition under PEDF treatment is contradictory with a possible function in PEDF-induced phagocytosis [70]. To go one step further in our study, we are now proposing to optimize the CRISPR-Cas9 genome editing technology to knock-out CD47, PNPLA2 and ATP5B in tumor and macrophage cells.

Seeking new therapeutic strategies, our findings on the P18 synthetic peptide is of high interest. This peptide has been previously described by the group of Dr. Volpert as carrying the functional epitope for PEDF anti-angiogenic and anti-tumor activities [38]. In the present study, we validated the same epitope as responsible for PEDF inflammatory function reemphasizing the great potential of this peptide as novel therapeutic target for PCa. Combination with immunotherapies that boost the activity of immune cells other than macrophages may also be interesting to test. One method could be to block the so-called checkpoint molecules—cytotoxic T lymphocyte–associated antigen 4 and programmed death 1—that inhibit T cells of the immune system [71]. As well, our results showing that PEDF can reprogram BMDMs (tumor-bearing mice) into M1 macrophages are very important as M1 reprogramming is currently recognized as the macrophages targeting therapy with the most promise [46].

While our model has been established *in vitro*, *in vivo* validation is now required with a special emphasis on the P18 peptide or P18-derived peptides as novel therapeutic agents. Still several unanswered questions/obscure points remain. 1) The precise signaling involved in macrophages polarization and tumor cell phagocytosis is uncharacterized and we are currently actively investigating these pathways. 2) The molecular mechanisms involved in tumor cell apoptosis are only partially identified. While we have demonstrated that PEDF induced the production of superoxide by macrophages concomitantly to CM-PEDF from macrophages stimulating tumor cell apoptosis, other secreted factors may be involved. Identifying these factors could explain the delay in PEDF-stimulated CD47 mRNA down-regulation (Fig 7E). As well, cell-cell contact may be involved and should be further investigated. 3) While we believe CD47 down-regulation by PEDF to be the key initiator of tumor cell phagocytosis, the participation of SIRPα is not totally proven. SIRPα inhibition by PEDF would support its involvement. On the other hand, the capacity in dissociating the interaction between CD47 and SIRPα has been described as more important than SIRPα expression level to regulate phagocytosis [41, 48]. The participation of other ligand such as SIRPγ, even though its affinity for CD47 is 10 times lower than SIRPα [72], is another possibility. Other previously identified ligand for CD47 such as thrombospondin-1 (TSP1) may also participate. As well, different “don’t eat me” signal such as calreticulin (CRP)-LRP1 (LDL receptor-related protein-1/CD91/α2-macroglobulin receptor) could be involved. Macrophage phagocytosis of apoptotic cells, or unopsonized viable CD47(-/-) red blood cells, has been attributed to the interaction between CRP on the target cell and LRP1 on the macrophage [73]. TSP1 interaction with CRT enhances the binding of CRT to LRP1 to stimulate focal adhesion disassembly leading subsequently to an intermediate adhesive phenotype necessary for cell growth, differentiation, migration, or survival [74]. Accordingly, TSP1 connects the separate cell-surface CRT, LRP1 and CD47 receptors functionally to regulate T-Lymphocyte cell adhesion [75]. On the other hand, TCR and CD28-mediated modulatory action on TSP-1 and LRP1 have been demonstrated to participate in antigen-induced reduction in T-cell motility, enhancement of contacts with antigen-presenting cells and activation; defined pre-requisites for the stimulation of an optimal adaptive immune response [76]. Further investigations are therefore needed to determine if PEDF and its effectors could be also involved in cell-mediated immunity.

## Conclusion

Our data introduce the novel concept that by targeting the tumor cell and macrophage molecular interactions, PEDF could enhance the tumoricidal activity of macrophages therefore extending the therapeutic role of PEDF in PCa.

## Supporting information

S1 Fig Protocol scheme for phagocytosis quantification. RAW 264.7 macrophages were cultured with CL1-Ctrl cells (Red) with or without PEDF (10 nM). Cells were imaged using Nomarski and Confocal microscopy (**Left panels**). Regions of interest (ROIs) were selected (Inset), and the intensity surface plot function (NIS-Elements AR 4.00.03) was used to measure the signal intensity (**Right panels**) of each ROI. ROI mean intensity from >30 selected ROIs was then calculated and data were showed using a boxplot graph from the IBM SPSS Statistics 23 software (as represented in Figs 2A, 3B, 8A, 9A and 10).
(TIF)

S2 Fig Spectral imaging microscopy of fluorescence in RAW 264.7 macrophages and CL1 tumor cell co-cultures. RAW 264.7 macrophages and CL1-Ctrl tumor cells (Fluorescent) co-cultures were imaged using the 545 nm narrow bandpass excitation filter and the 570 nm long bandpass dichroic mirror. A halogen lamp was used to obtain zero order spectra (**A**). Cells were imaged using a 60x oil objective (N.A 1.4) and a 500 msec exposure. Image **B** was obtained using a 200 μm slit width on the spectrograph. For image **C**, the slit width was successfully closed to 100 μm. Vertical dashed lines: representation of the final slit width as showed on image **D**. For image **D**, the slit width was closed to 0.5 μm. This ensured the highest spatial resolution from a discrete area. The areas corresponding to the CL-1 cytoplasm, phagosome in macrophages (RAW 264.7) and background (inter-cellular space) were obtained from regions of interest (ROI) as indicated by the arrows. ~100 regions in each CL1-Ctrl and RAW 264.7 cells were analyzed per experiment. The spectral outputs of the fluorescence in a macrophage phagosome and a neighboring cancer cell were concomitantly analyzed. Additionally, ROIs selected in the inter-cellular space in co-culture and RAW 264.7 mono-culture were used to set up baseline. The fluorescence data was converted to ASCII format, prior to analysis with SigmaPlot (version 8.0). Two experiments with similar results obtained were performed.
(TIF)

S3 Fig PEDF expression stimulates the migration of RAW 264.7 cells towards 2D conventional prostate tumor cell mono-culture *in vitro*. RAW 264.7 macrophage chemotaxis to PC3-Ctrl, PC3-PEDF, CL1-Ctrl, and CL1-PEDF cells was tested using the Inverted Boyden chamber assay as we previously described in [33]. The data were normalized as the percentage of maximal migration [PC3-Ctrl- and CL1-Ctrl-induced migration taken for 100%]. PEDF specificity on macrophages migration was validated by neutralization assays using PEDF-specific blocking (MAB1059, 5μg/ml) or isotype antibodies. The migration was counted in 10 high-powered fields per condition, each condition tested in quadruplicate and the experiments done at least thrice. *: p < 0.05 is shown to illustrate statistical significance.
(TIF)

S4 Fig Production of Superoxide Radicals in RAW 264.7 cells using the WST-1 Tetrazolium-based *in vitro* assay. RAW 264.7 macrophages were treated for 48 hours ± PEDF (10 nM). Formazan production to quantitatively estimate the Superoxide radical production was then measured using the WST-1 kit (Sigma-Aldrich). Data points represent mean ± SD of quadruplicate samples from two independent experiments. Statistical analyses were performed using the Student’s t test, *: p < 0.05.
(TIF)

S5 Fig Phagocytosis visualization in PCa/RAW 264.7 co-cultures treated with PNPLA2 and ATP5B inhibitors. Representative Nomarski/Confocal images (Left panels) of PCa cell phagocytosis in CL1-Ctrl (Red)/RAW 264.7 co-cultures treated with α-CD47 (100ng/μl; Green), PEDF or P18 (10 nM) alone or PEDF/P18 in combination with either S-BEL (5μM) or Angiostatin (10 nM). Inset: representative ROI selected for quantification using the intensity surface plot function (NIS-Elements AR 4.00.03).
(TIF)

S6 Fig mRNAs expression levels of Angiostatin receptors in RAW 264.7 cells. Total RNAs from RAW 264.7 cells treated with PEDF (10 nM), Angiostatin (10 nM), or combination were analyzed by qRT-PCR for Angiostatin receptors (Annexin A2 # 330001 PPM34399F, c-Met # 330001 PPM03726A, Integrin beta 3 # 330001 PPM03687E, and Integrin alpha V # 330001 PPM03662D; all from Qiagen) and normalized to S15. Results are presented as relative fold change compared to control non-treated cells. Data points represent mean ± SD of triplicate samples from two independent experiments.
(TIF)
