# Supplementary figures and images for: PEDF increases the tumoricidal activity of macrophages towards prostate cancer cells in vitro
Source: PLoS One. 2017 Apr 12;12(4):e0174968. doi: 10.1371/journal.pone.0174968 (PMC5389654; doi:10.1371/journal.pone.0174968)

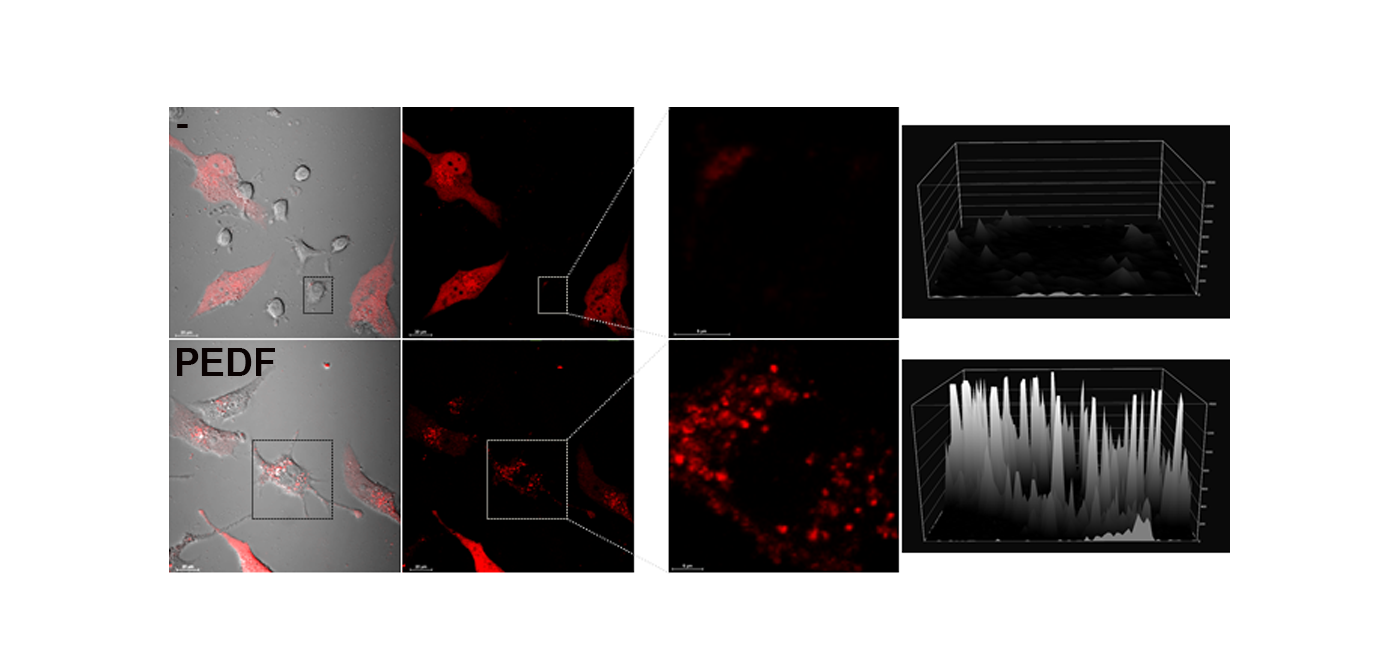

Supplement: S1 Fig — RAW 264.7 macrophages were cultured with CL1-Ctrl cells (Red) with or without PEDF (10 nM). Cells were imaged using Nomarski and Confocal microscopy (Left panels). Regions of interest (ROIs) were selected (Inset), and the intensity surface plot function (NIS-Elements AR 4.00.03) was used to measure the signal intensity (Right panels) of each ROI. ROI mean intensity from >30 selected ROIs was then calculated and data were showed using a boxplot graph from the IBM SPSS Statistics 23 software (as represented in Figs 2A, 3B, 8A, 9A and 10). (TIF) [file pone.0174968.s001.tif]

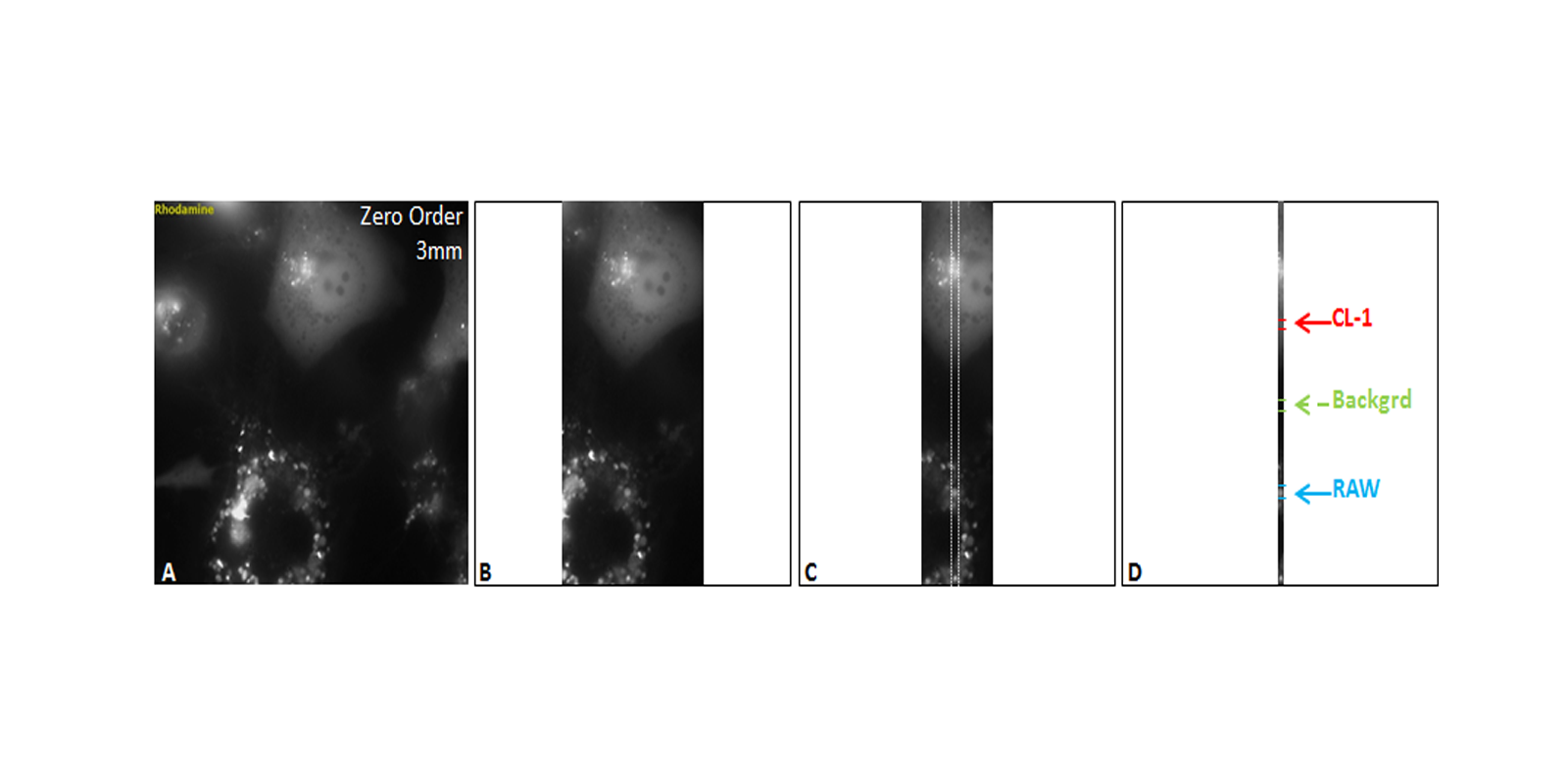

Supplement: S2 Fig — RAW 264.7 macrophages and CL1-Ctrl tumor cells (Fluorescent) co-cultures were imaged using the 545 nm narrow bandpass excitation filter and the 570 nm long bandpass dichroic mirror. A halogen lamp was used to obtain zero order spectra (A). Cells were imaged using a 60x oil objective (N.A 1.4) and a 500 msec exposure. Image B was obtained using a 200 μm slit width on the spectrograph. For image C, the slit width was successfully closed to 100 μm. Vertical dashed lines: representation of the final slit width as showed on image D. For image D, the slit width was closed to 0.5 μm. This ensured the highest spatial resolution from a discrete area. The areas corresponding to the CL-1 cytoplasm, phagosome in macrophages (RAW 264.7) and background (inter-cellular space) were obtained from regions of interest (ROI) as indicated by the arrows. ~100 regions in each CL1-Ctrl and RAW 264.7 cells were analyzed per experiment. The spectral outputs of the fluorescence in a macrophage phagosome and a neighboring cancer cell were concomitantly analyzed. Additionally, ROIs selected in the inter-cellular space in co-culture and RAW 264.7 mono-culture were used to set up baseline. The fluorescence data was converted to ASCII format, prior to analysis with SigmaPlot (version 8.0). Two experiments with similar results obtained were performed. (TIF) [file pone.0174968.s002.tif]

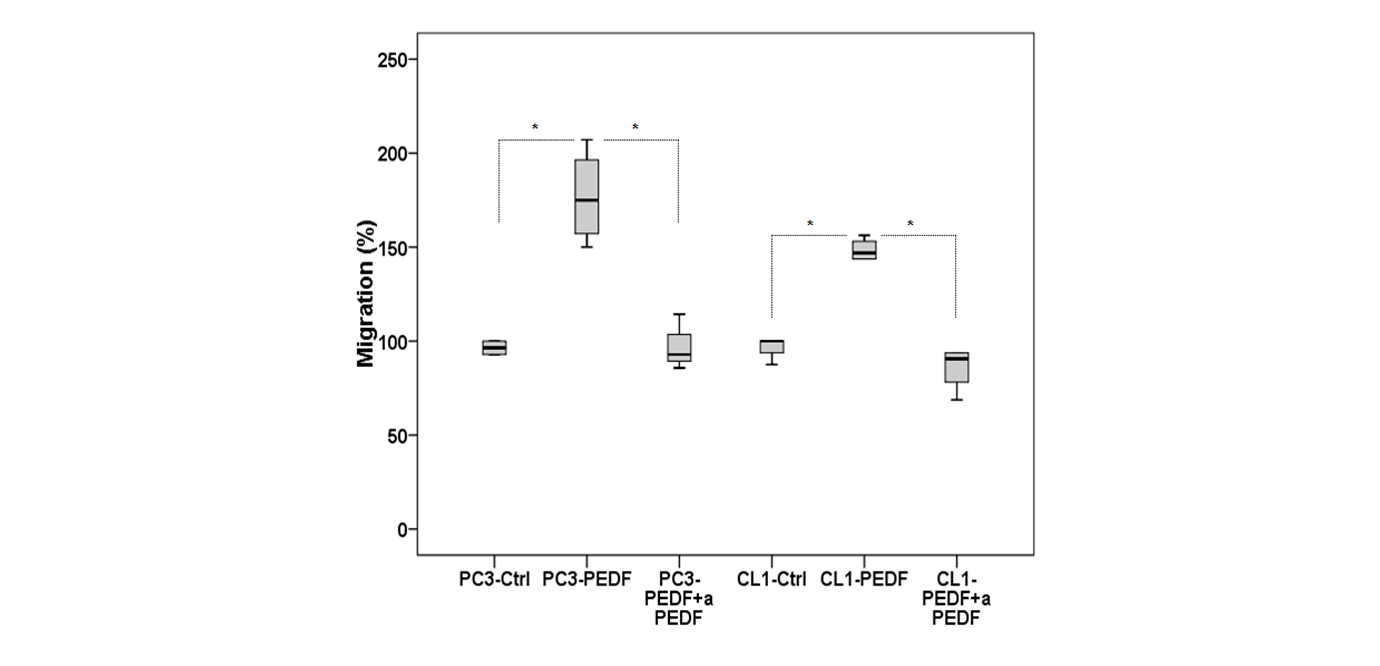

Supplement: S3 Fig — RAW 264.7 macrophage chemotaxis to PC3-Ctrl, PC3-PEDF, CL1-Ctrl, and CL1-PEDF cells was tested using the Inverted Boyden chamber assay as we previously described in [33]. The data were normalized as the percentage of maximal migration [PC3-Ctrl- and CL1-Ctrl-induced migration taken for 100%]. PEDF specificity on macrophages migration was validated by neutralization assays using PEDF-specific blocking (MAB1059, 5μg/ml) or isotype antibodies. The migration was counted in 10 high-powered fields per condition, each condition tested in quadruplicate and the experiments done at least thrice. *: p < 0.05 is shown to illustrate statistical significance. (TIF) [file pone.0174968.s003.tif]

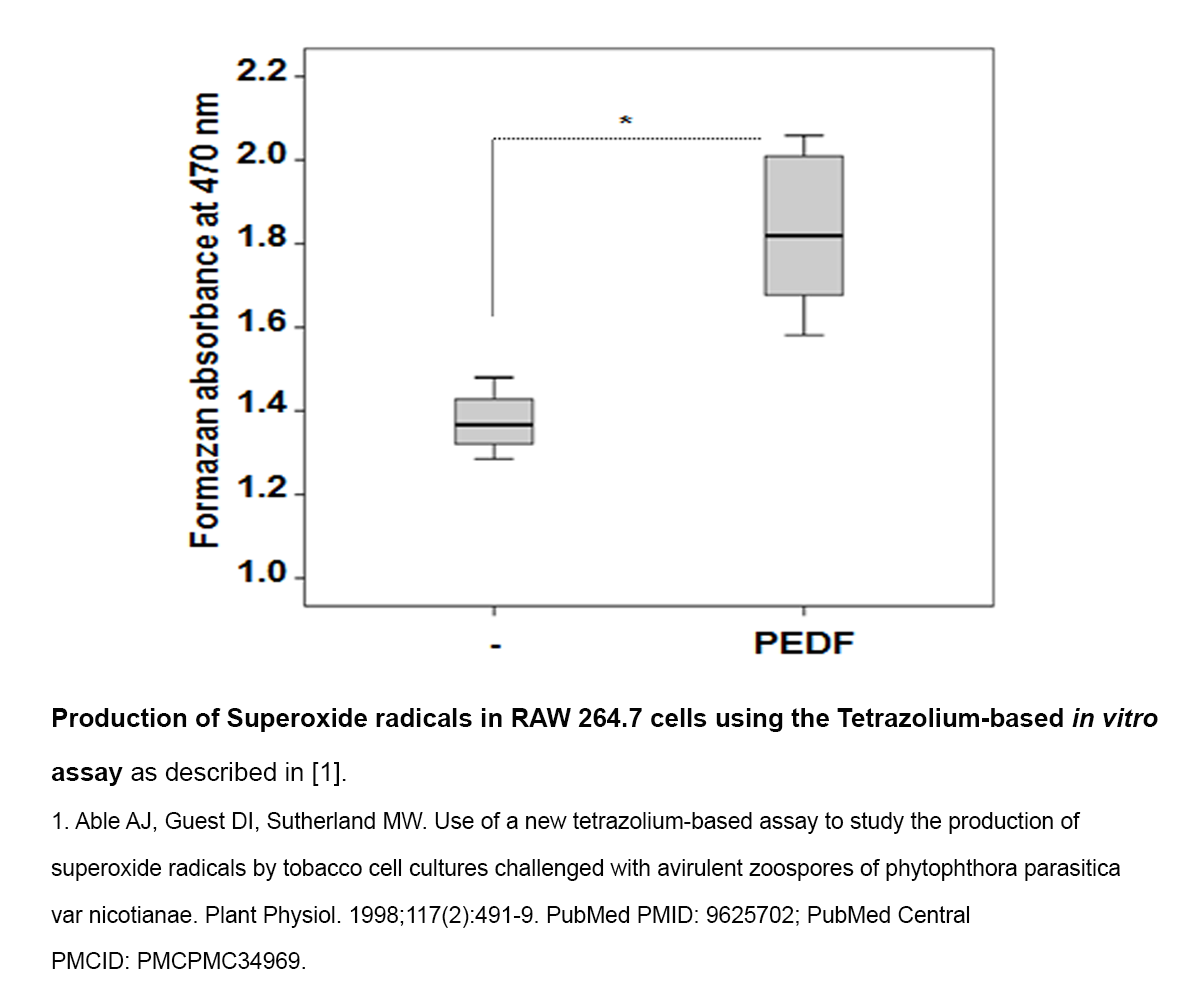

Supplement: S4 Fig — RAW 264.7 macrophages were treated for 48 hours ± PEDF (10 nM). Formazan production to quantitatively estimate the Superoxide radical production was then measured using the WST-1 kit (Sigma-Aldrich). Data points represent mean ± SD of quadruplicate samples from two independent experiments. Statistical analyses were performed using the Student’s t test, *: p < 0.05. (TIF) [file pone.0174968.s004.tif]

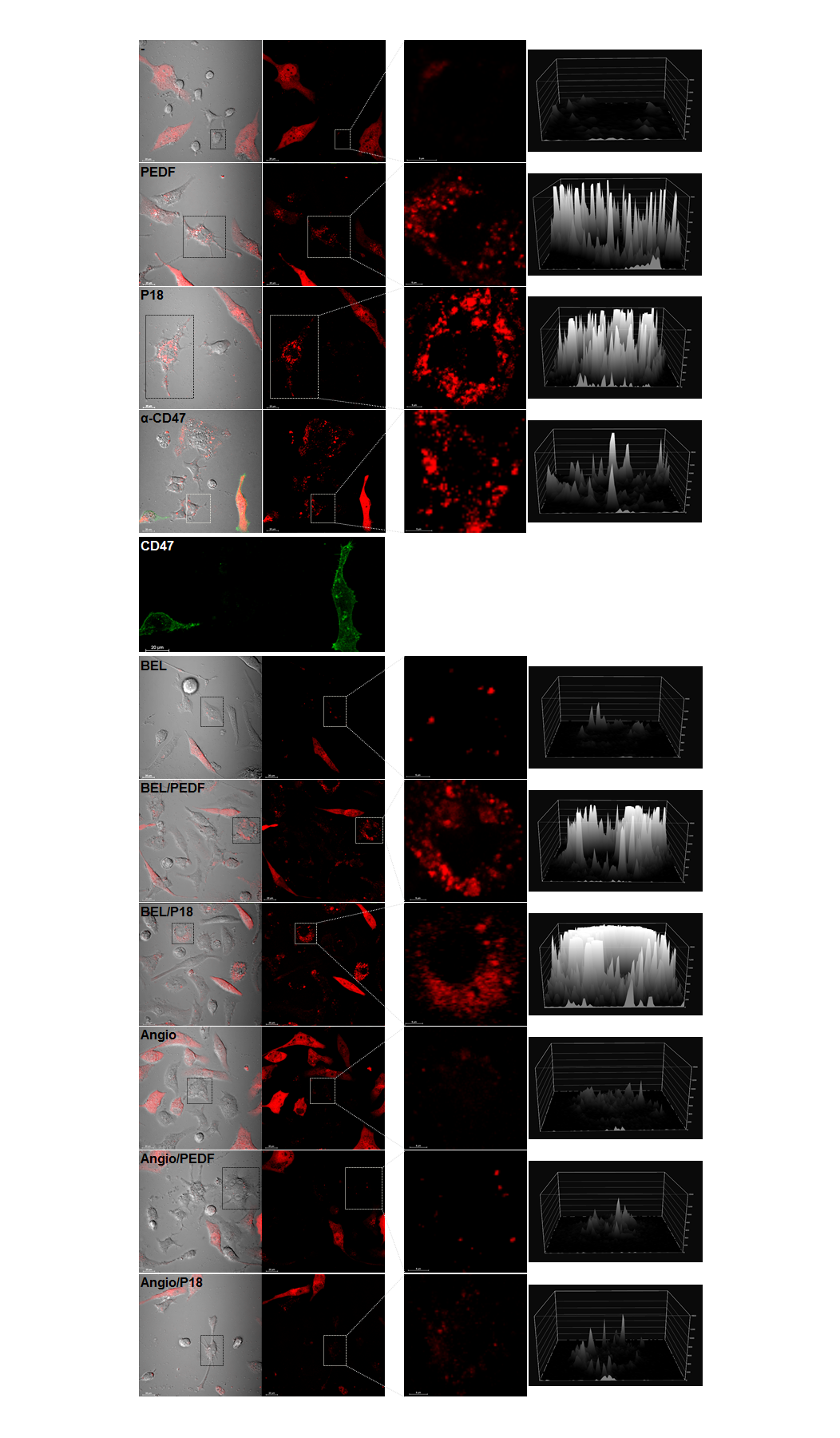

Supplement: S5 Fig — Representative Nomarski/Confocal images (Left panels) of PCa cell phagocytosis in CL1-Ctrl (Red)/RAW 264.7 co-cultures treated with α-CD47 (100ng/μl; Green), PEDF or P18 (10 nM) alone or PEDF/P18 in combination with either S-BEL (5μM) or Angiostatin (10 nM). Inset: representative ROI selected for quantification using the intensity surface plot function (NIS-Elements AR 4.00.03). (TIF) [file pone.0174968.s005.tif]

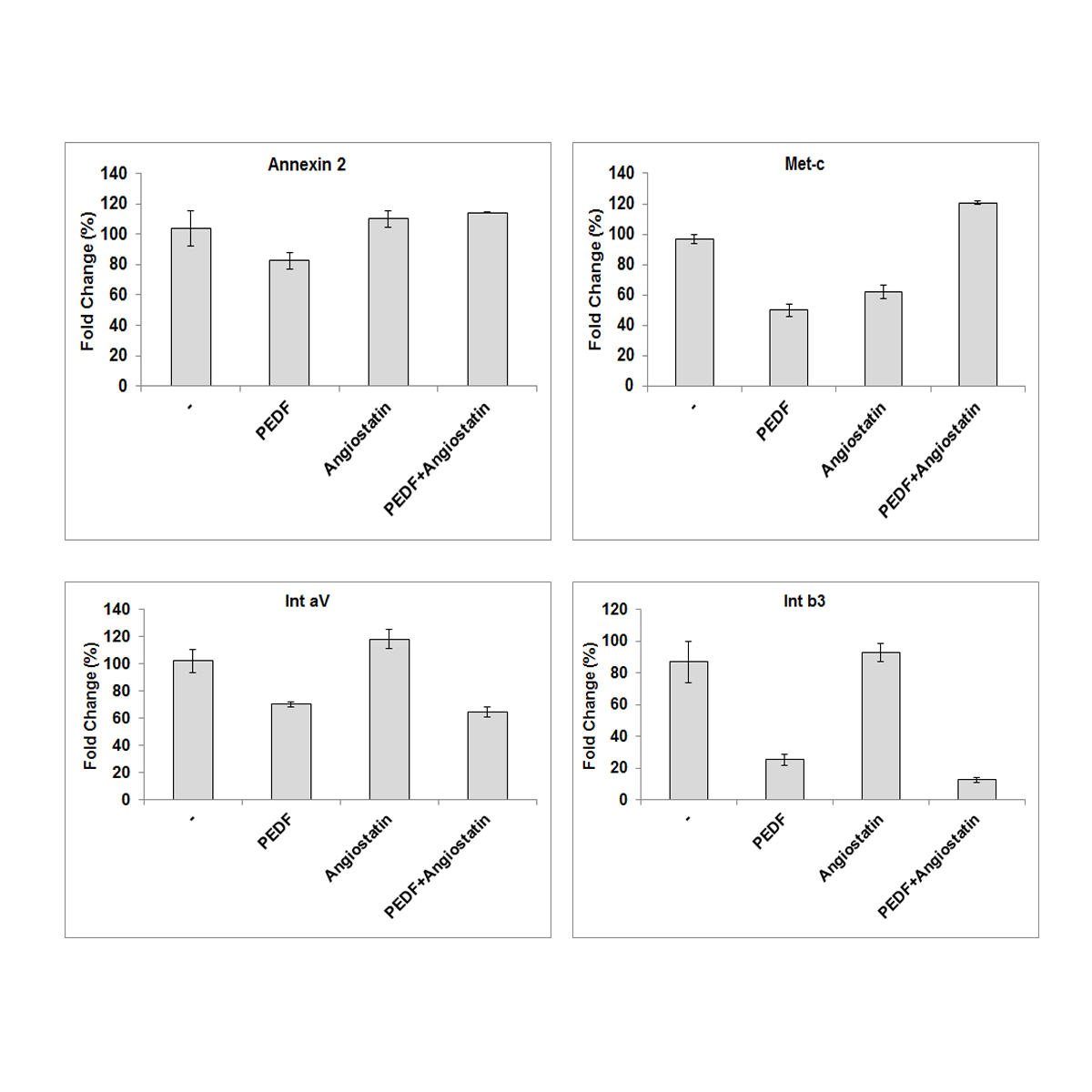

Supplement: S6 Fig — Total RNAs from RAW 264.7 cells treated with PEDF (10 nM), Angiostatin (10 nM), or combination were analyzed by qRT-PCR for Angiostatin receptors (Annexin A2 # 330001 PPM34399F, c-Met # 330001 PPM03726A, Integrin beta 3 # 330001 PPM03687E, and Integrin alpha V # 330001 PPM03662D; all from Qiagen) and normalized to S15. Results are presented as relative fold change compared to control non-treated cells. Data points represent mean ± SD of triplicate samples from two independent experiments. (TIF) [file pone.0174968.s006.tif]
